# Opsoclonus–myoclonus syndrome caused by varicella-zoster virus

**DOI:** 10.4103/0972-2327.70876

**Published:** 2010

**Authors:** Dilip Singh, Manish Sinha, Rajesh Kumar, Rakesh Shukla, R. C. Ahuja

**Affiliations:** Department of Neurology, Chhatrapati Shahuji Maharaj Medical University, Lucknow, U.P., India; 1 Department of Medicine, Chhatrapati Shahuji Maharaj Medical University, Lucknow, U.P., India

**Keywords:** Opsoclonus, myoclonus, varicella-zoster virus

## Abstract

Opsoclonus–myoclonus syndrome (OMS) is a rare condition that has been reported from all parts of the world. It is well recognized as a paraneoplastic syndrome in children with neuroblastoma and in adults with small-cell carcinoma of lung and some other cancers. It may also occur in association with various central nervous system infections. We report a case of OMS in a patient with varicella zoster virus infection. IgM antibody for varicella-zoster virus was detected in the serum and the cerebrospinal fluid. The patient improved after treatment with clonazepam and was asymptomatic at 1-month follow-up.

## Introduction

Opsoclonus–myoclonus syndrome (OMS) is seen in 1 in 10,000,000 persons per year. It was first described by Kinsbourne in 1962.[1] It affects 2% of children with neuroblastoma.[2] About half of all cases are associated with neuroblastoma and most of the others are suspected to be associated with a low-grade neuroblastoma that spontaneously regresses before detection. The remaining cases have been associated with a viral infection (St. Louis encephalitis, Epstein-Barr, coxsackie B, or enterovirus), though a direct connection has not been proven. Our literature search for OMS due to varicella-zoster was negative except for one case report in the Spanish language.[3] In this article we report a case of OMS due to varicella-zoster virus infection.

## Case Report

A 14-year-old girl presented with high-grade continuous fever without chills or rigors. The fever was associated with nausea and a few episodes of non-projectile, non-bilious vomiting. On the fifth day of fever she developed tremulousness of the whole body and a staggering gait.

At admission to the hospital the patient was febrile; her vital signs were normal. She was conscious, oriented, and could follow commands. She had involuntary, arrhythmic, high-amplitude, conjugate ocular movements in all directions. When asked to look at a target, her visual fixation was disrupted by bursts of high-frequency, conjugate ocular oscillations that had horizontal, vertical, and torsional components, suggestive of opsoclonus. She also had sudden brief involuntary jerky movements of her limbs, trunk, and head, suggestive of myoclonus. The myoclonic movements increased on sitting or standing. The rest of the neurological and systemic examination was normal, except for mild hepatosplenomegaly.

Investigations revealed hemoglobin: 9.7 gm/dl; total leukocyte count: 16.0 × 10^9^/l; differential leukocyte count: polymorphs 67%, lymphocytes 27%, monocytes 3%, and eosinophils 3%; and platelet count: 150 × 10^9^/l. Peripheral blood smear for malaria was negative. Typhoid IgM was negative. Serum sodium was 139 mmol/l, serum potassium 4.5 mmol/l, blood urea 27 mg/dl, serum creatinine 0.5 mg/dl, random blood sugar 91 mg/dl, serum bilirubin 1.2 mg/dl, serum alkaline phosphatase 1095 IU/l, SGPT 103 IU/l, serum proteins 6.6 gm/dl, and serum albumin 3 gm/dl. Examination of the cerebrospinal fluid revealed proteins 49.4 mg/dl, and sugar 49.8 mg/dl (corresponding blood sugar was 127.4 mg/dl); total cell count was 15/mm^3^ (polymorphs 30% and lymphocytes 70%). Gram’s stain and AFB stain were negative. Varicella-zoster IgM antibody titers were positive in the CSF and serum by ELISA, with rising titers over 4 weeks. Epstein-Bar virus, coxsackie A and B, Japanese encephalitis, and enterovirus 68–71 IgM antibody titers were negative in the CSF and serum. A 1.5-T magnetic resonance imaging of brain, which included T1W, T2W and post-gadolinium contrast images, revealed no abnormality. Plain x-ray of the chest was normal. Ultrasound abdomen revealed mild hepatosplenomegaly.

The patient was diagnosed as a case of opsoclonus–myoclonus syndrome with varicella-zoster infection. She was treated with clonazepam 0.5 mg thrice a day, which was gradually increased to 1 mg thrice a day. The opsoclonus and myoclonus completely disappeared over the next 15 days. The clonazepam dose was tapered from the fifteenth day onwards by 0.5 mg per day. At 1-month follow-up the patient was asymptomatic.

## Discussion

Our patient was diagnosed as having OMS caused by varicella-zoster virus infection; however, acyclovir was not used as there was no sign of active infection, and the diagnosis was based on demonstration of rising antibody titers in the serum and CSF. Ultrasound abdomen and x-ray chest were normal, thus helping to rule out the neoplastic conditions commonly associated with OMS. Further paraneoplastic screening, including estimation of onconeuronal antibodies was not performed as the patient showed remarkable improvement with clonazepam.

The term ‘opsoclonus’ is defined as chaotic, conjugate, multivector, back-to-back, saccadic eye movements without intersaccadic latency. Although the exact pathophysiology of opsoclonus remains unclear, findings of recent pathological and functional MRI studies suggest that disinhibition of the fastigial nucleus of the cerebellum is involved. The classic concept of damage to the omnipause cells in the pontine raphe is not supported by autopsy studies.[4] Opsoclonus is often precipitated or exacerbated by blinking or eyelid closure, both of which may suppress omnipause neurons. OMS is encountered in patients with encephalitis, in association with certain neoplasms (notably, neuroblastoma in children and gynecological cancers in adults), and certain toxins. The onconeuronal antibodies associated with OMS are anti-Ri antibodies (gynecologic cancers) and, less frequently, anti-Hu, anti-Yo, and anti-Ma-2 antibodies.[4] Epstein-Barr virus, coxsackie virus, and enterovirus have been incriminated in OMS.[5,6]

The various therapies that have been tried successfully are corticosteroids, intravenous immunoglobulins, immuno-suppressants, plasmapheresis, rituximab, ACTH, clonazepam, baclofen, valproate, 5-hydroxytryptophan, etc. In the present case, a significant improvement was observed with clonazepam over a short period of time. Clonazepam might be a very useful drug to treat these symptoms in patients of OMS, especially where an infectious etiology is suspected, as the use of steroids can result in flaring up of infection in such patients. The main action of clonazepam is its ability to interact with benzodiazepine receptors in the brain that facilitate inhibitory GABAergic transmission. The drug is also cost-effective, easily available, and free of serious side effects when used for a short duration.

The present report suggests that varicella-zoster may be added to the list of viruses which can cause OMS. A positive viral serology may obviate the need for estimation of onconeural antibodies. The condition can be easily treated with clonazepam.
